# Brain function distinguishes female carriers and non-carriers of familial risk for autism

**DOI:** 10.1186/s13229-020-00381-y

**Published:** 2020-10-20

**Authors:** Adam T. Eggebrecht, Ally Dworetsky, Zoë Hawks, Rebecca Coalson, Babatunde Adeyemo, Savannah Davis, Daniel Gray, Alana McMichael, Steven E. Petersen, John N. Constantino, John R. Pruett

**Affiliations:** 1 Mallinckrodt Institute of Radiology, Washington University School of Medicine, 660 S. Euclid Ave, St Louis, MO 63110 USA; 2 Department of Neurology, Washington University School of Medicine, 660 S. Euclid Ave, St Louis, MO 63110 USA; 3 Department of Psychological and Brain Sciences, Washington University in St. Louis, 1 Brookings Dr., St Louis, MO 63130 USA; 4 Department of Psychiatry, Washington University School of Medicine, 660 S. Euclid Ave, St Louis, MO 63110 USA; 5 Washington University School of Medicine, C.B. 8225, 4515 McKinley Ave., St. Louis, MO 63110 USA

**Keywords:** Sex ratio, Endophenotype, Biological motion, Silent transmission, Familial risk

## Abstract

**Background:**

Autism spectrum disorder (ASD) is characterized by high population-level heritability and a three-to-one male-to-female ratio that occurs independent of sex linkage. Prior research in a mixed-sex pediatric sample identified neural signatures of familial risk elicited by passive viewing of point light motion displays, suggesting the possibility that both resilience and risk of autism might be associated with brain responses to biological motion. To confirm a relationship between these signatures and inherited risk of autism, we tested them in families enriched for genetic loading through undiagnosed (“carrier”) females.

**Methods:**

Using functional magnetic resonance imaging, we examined brain responses to passive viewing of point light displays—depicting biological versus non-biological motion—in a sample of undiagnosed adult females enriched for inherited susceptibility to ASD on the basis of affectation in their respective family pedigrees. Brain responses in carrier females were compared to responses in age-, SRS-, and IQ-matched non-carrier-females—i.e., females unrelated to individuals with ASD. We conducted a hypothesis-driven analysis focused on previously published regions of interest as well as exploratory, brain-wide analyses designed to characterize more fully the rich responses to this paradigm.

**Results:**

We observed robust responses to biological motion. Notwithstanding, the 12 regions implicated by prior research did not exhibit the hypothesized interaction between group (carriers vs. controls) and point light displays (biological vs. non-biological motion). Exploratory, brain-wide analyses identified this interaction in three novel regions. Post hoc analyses additionally revealed significant variations in the time course of brain activation in 20 regions spanning occipital and temporal cortex, indicating group differences in response to point light displays (irrespective of the nature of motion) for exploration in future studies.

**Limitations:**

We were unable to successfully eye-track all participants, which prevented us from being able to control for potential differences in eye gaze position.

**Conclusions:**

These methods confirmed pronounced neural signatures that differentiate brain responses to biological and scrambled motion. Our sample of undiagnosed females enriched for family genetic loading enabled discovery of numerous contrasts between carriers and non-carriers of risk of ASD that may index variations in visual attention and motion processing related to genetic susceptibility and inform our understanding of mechanisms incurred by inherited liability for ASD.

## Background

Autism spectrum disorder (ASD) is a highly heritable, neurodevelopmental disorder characterized by deficits in social communication and interaction as well as restricted interests and repetitive behaviors. Overwhelming evidence points to a substantial genetic influence on the total population burden of ASD [1–4], for which the heritability has been estimated at 0.80 or higher [5–7]. The causation of ASD has been traced to myriad genetic mechanisms, including the deleterious effects of both common and individually rare, highly penetrant mutations [8]. Additionally, the sibling recurrence rate of ASD is 10–18% and over half of the genetic liability to ASD is estimated to arise from polygenic risk [9, 10]. One important characteristic of ASD is the striking 3:1 male/female sex ratio [10, 11] that has been observed both across and within families affected by ASD. This sexual dimorphism is especially remarkable given that the extant genetic variants implicated in ASD are overwhelmingly autosomal and involve multiple distinct regions of the genome, and the sets of genetic susceptibility factors associated with ASD in males lack consistent differences with those in females with ASD [12–14].

Substantial genetic-epidemiologic evidence shows that inherited liability for ASD is commonly transmitted to (and through) females who appear entirely unaffected or exhibit phenotypes that are substantially muted compared to those of their ASD-affected male relatives [1, 3, 9, 15]. In other words, differential phenotypic expression occurs in the context of what is believed to be an equivalent inherited liability for ASD between males and females in the population [16]. The mechanisms by which penetrance varies by sex across diverse autosomal causes of ASD liability remain unknown [12, 17–20]. Such sex differences are commonly attributed to a “female protective effect” (FPE [18]), although research evidence suggests they may more aptly be ascribed to enhanced sensitivity among males [16]. Understanding the mechanism(s) by which genetic liability for ASD can be carried by (and transmitted through) unaffected individuals represents an important scientific frontier in brain and behavioral research. Characterizing that effect would represent a significant prospect for higher impact intervention, particularly among males who are disproportionately influenced by genetic susceptibility.

This study was further motivated by the fact that, in spite of the pronounced heritability of ASD, *most* affected children are born to unaffected parents. Only a minority of cases can be accounted for by de novo genetic variation, and such variants can never be invoked as the sole cause of ASD when it recurs in a family, which is common. Currently, there is no way to predict whether transmission of ASD through the close relative (e.g., an unaffected sibling) of an affected individual might occur. To this end, the present study explored whether candidate neural signatures previously reported among close relatives of individuals with ASD might serve as indicators of transmission risk. It is known that there are average elevations of subclinical autistic trait scores among relatives of individuals with ASD within and across generations [15]. However, the magnitude and variability of these elevations render them insufficient for individual prediction of ASD risk to offspring.

Although individuals with ASD exhibit a readily identifiable, often severely impairing behavioral phenotype, extensive studies of brain structure and function have generally failed to confirm replicable neural signatures of autistic impairment [21–23]. Nevertheless, focused studies of brain morphology, activation, and connectivity involving social brain circuitry have begun to reveal key contrasts between carefully selected subgroups of affected individuals (e.g., ASD in Fragile × Syndrome) and typically developing controls that partially overlap with known sexual dimorphisms observed in brain development in typically developing individuals [24, 25]. This study was designed to determine whether neural signatures that have been proposed as ASD endophenotypes [26] might be traceable in a sample of undiagnosed women substantially enriched (on average) for inherited susceptibility to ASD, i.e. on the basis of family pedigree information, typically inferring transmission of ASD from an affected first degree relative through a mother to her affected offspring. Selection for enhanced family genetic loading in this manner allows for enrichment of asymptomatic carriage of inherited susceptibility, which cannot yet be assigned with confidence on the basis of measured polygenic risk. We know of no other published attempt to image a sample of individuals enriched for ASD susceptibility to this degree and who were simultaneously (by virtue of being nevertheless unaffected by ASD) able to be matched to typically developing controls for level of social functioning, cognition, and key aspects of behavioral variation.

To date, one of the most compelling neural read-outs of endophenotypic liability for ASD has involved patterns of brain activation in response to viewing point light displays of biological motion [26]. Visual sensitivity to biological motion is an evolutionarily conserved mechanism that is fundamental to adaptive social engagement [27] is believed critical for filial attachment [28] and argued by some to be important for the attribution of intentions to others [29]. Reduced response to biological motion stimuli in children with ASD has been widely noted and is thought to be associated with the dysregulation of appropriate social behavior [30–32]. In fact, normative visual engagement to faces, biological motion, and dynamic social scenes has been shown to shape typical infant development from birth [33–35] and is strongly influenced by genetic factors [36]. A prior neuroimaging study identified three unique profiles of group contrasts when comparing ASD children, their unaffected siblings (US), and a group of unrelated typically developing children (TD) with respect to brain activation in response to viewing point-light movies of biological motion [26]. First, “state” activity identified reduced activation to biological motion specific only to the ASD group when compared to US and TD. Second, there was evidence of “trait” activity, where both the ASD and US children displayed reduced response to the stimuli, indicating a predisposition to developing ASD in comparison with the TD population. Finally, among the US children, there was significant activation in specific regions that were not identified in either the ASD or TD groups. The existence of this signal in the unaffected siblings was hypothesized to “compensate” for a greater genetic risk of developing ASD.

Herein, we aimed to investigate these neural signatures of ASD in a carefully selected set of females suspected of carrying and/or silently transmitting genetic susceptibility to ASD—all first-degree relatives of affected index cases. We compared them to a sample of females with no known genetic liability for ASD who were matched for age, cognitive ability, social function and other aspects of behavioral variation. We measured brain activations with functional magnetic resonance imaging (fMRI), while participants viewed silent video clips containing point-light displays of biological or scrambled motion. We hypothesized that previously described “compensatory” brain regions would exhibit stronger differential activity for biological vs. scrambled motion in females suspected of carrying genetic susceptibility for ASD relative to females without known genetic risk. We also hypothesized that previously described “trait” brain regions would exhibit weaker differential activity for biological vs. scrambled motion in females suspected of carrying genetic susceptibility for ASD relative to females without known genetic risk. We also conducted additional hypothesis-generating, exploratory brain-wide analyses to investigate more fully potential neural signatures relating to an otherwise silent transmission of heightened genetic risk of ASD.

## Methods

### Participants

Adult carrier females (CF) were individuals unaffected by clinically diagnosed ASD but with strong evidence of carrying or transmitting inherited liability. Carrier females were identified on the basis of specific patterns of familial aggregation of ASD (Additional file 1: Supplementary Fig. 1), representing a range of elevations over the population average: from women with affected first degree relatives in the same generation to mothers of concordant ASD-affected maternal half-siblings. Adult non-carrier females (NCF) were individuals (1) *not* affected by clinical ASD, (2) *not* related to a first- or second-degree relative with ASD, and (3) *with* quantitative autistic trait scores distributed across the lower four quintiles of the general population distribution for females. Quantitative autistic traits were ascertained with the Social Responsiveness Scale—2 (SRS; see below). Importantly, quantitative autistic trait scores were matched between CF and NCF groups. The selection for enrichment of female carrier status predominantly included pedigrees featuring silent maternal transmission to offspring; thus all but one of the carrier females was a mother (see pedigree diagrams in Additional file 1: Supplementary Materials), and all but two females in the NCF group were mothers. Non-carrier females were recruited from the community, and CF were recruited from both the local community and the Washington University Social Developmental Studies program of one of the senior authors. The research protocol was approved by the institutional review board at Washington University School of Medicine (WUSM), and participants provided written consent after receiving a detailed description of the study. Behavioral assessments and MRI data were acquired at WUSM, and the data were used for research purposes only. All participants had normal or corrected-to-normal vision via MRI-compatible lenses.

### Inclusion criteria

Participants selected for inclusion in the CF group were women who did not have clinically diagnosed ASD, who had a family pedigree consistent with familial loading for genetic susceptibility to ASD, ranging from a minimum of a single male first degree relative affected, to complex multigenerational pedigrees in which ASD would be suspected to have been transmitted through the participant (see Additional file 1: Supplementary Fig. 1). The CF participants were asked to review their family pedigree with a research staff member and identify first and second degree relatives diagnosed or suspected of having ASD. For those identified as suspected but not diagnosed, the participant completed SRS ratings on the individual (those with scores that exceeded the lower boundary of the scale’s published clinical range are represented in gray in the pedigree diagrams in Additional file 1: Supplementary Fig. 1). NCF were selected based on not having a diagnosis of ASD in addition to not having any first- or second-degree relatives with a diagnosis or suspected case of ASD.

### Behavioral measures

Behavioral phenotyping of both the CF and NCF groups included the Social Responsiveness Scale (SRS-2 Adult Self Report version [37]), the Adult Behavior Checklist (ABCL Adult Self Report version [38]), and the Raven's Progressive Matrices [39] by a trained clinician. The CF group also completed an age appropriate version of the SRS-2 and ABCL (or Child Behavioral Checklist) on each first degree relative diagnosed or suspected of autism.

### Image acquisition

Neuroimaging consisted of structural, task-based functional MRI, diffusion-weighted MRI, and resting state functional brain MRI. All anatomical and functional images were acquired on a Siemens 3T Prisma MRI scanner using a 20-channel head coil. (A 32-channel coil was also piloted but was not selected due to being incompatible with the use of our eye tracker. We attempted to collect eye tracking data in these cohorts with the 20-channel head coil but are not presenting the data here due to poor data quality.) For each participant, a T1-weighted sagittal MPRAGE image (208 slices with 0.8 mm voxels, TE = 2.22 ms, TR = 2.4 s, flip angle = 8°) and a T2-weighted sagittal image (208 slices with 0.8 mm voxels, TE = 563 ms, TR = 3.2 s, flip angle = 120°) were collected. A scanning session included six runs of functional MRI scanning: four runs of resting-state fMRI and two runs of the point-light task, with each run lasting 8.1 min. All functional images were obtained using a BOLD gradient-echo echo-planar sequence (TR = 1.16 with a multi-band factor of 4, TE = 32.4 ms, flip angle = 63 degrees, 64 slices with 2.4 mm voxels). A gradient echo field mapping sequence and DBSI data were collected in each session but were not used in the present analysis. Additionally, the resting state data are to be presented elsewhere.

Each fMRI run involved imaging of the brain response to point light displays (PLDs). Point light stimuli were identical to those used by Kaiser et al. in their 2010 paper [26]. In the present study, participants viewed 24-s silent video clips containing PLDs of biological or scrambled motion presented at a video frame rate of 30 frames per second. Twelve biological and scrambled motion clips (6 of each condition) were displayed in an alternating block design (cf. [26, 40]), with the exception of 24-s fixation periods between each PLD movie and before and after stimulus presentation. Participants were instructed simply to attend to the videos throughout the experiment. The procedure required approximately eight minutes of time per PLD run. Stimuli were presented via the Psychophysics Toolbox-3 MATLAB software package.

### fMRI preprocessing

Data from all functional images were preprocessed to remove noise and artifacts (refer to previous studies [41] for detailed procedures). Briefly, for each session, sinc interpolation was performed to correct for temporal misalignment in acquisition across slices, whole brain intensity within each BOLD run was normalized to achieve a mode value of 1000, and movement correction was performed within and across runs by a rigid body realignment process. Each subject’s functional data were transformed into the stereotactic Talairach atlas space [42] and resampled to 2-mm isotropic voxels. Any BOLD runs with a root-mean-square framewise displacement (FD) [43] of less than 1.5 mm were retained for analyses.

### Demographics

All data sets were subject to stringent MRI quality control criteria. A total of 29 CF (age range 25–64 years) and 28 NCF (age range 24–59 years) were scanned with the protocol. One CF was excluded due to falling asleep in the scanner. From the remaining 28 CF and 28 NCF, participants were removed to match for SRS across groups: two because SRS scores were not collected, five CF because they had high scores (above 55), and three NCF with low scores (below 40). A total of 21 CF and 25 NCF successfully completed all of the behavioral measures and the fMRI imaging session with RMS movement < 1.5 mm during each fMRI run. In the participants of focus, age did not differ between groups: *t* = 0.667, *p* = 0.508, d.o.f. = 44, *d* = 0.197. Mean scores on the SRS-2 did not differ between groups (CF: *n* = 21, range: 40–53, mean (standard deviation): 45.0 (3.7); NCF: *n* = 25, range: 40–54, mean (standard deviation): 43.7 (3.8); one-tailed *t* = 1.198, *p* = 0.119, d.o.f. = 44, *d* = 0.355). The groups also did not differ in IQ (CF: *n* = 21, range: 79–133, mean (standard deviation): 101.0 (15.0); NCF: *n* = 25, range: 80–133, mean (standard deviation): 100.3 (13.1); one-tailed *t* = 0.119, *p* = 0.906, d.o.f. = 44. The distribution of SRS-2 scores for the group of "suspected" relatives among the pedigrees of CF (Additional file 1: Supplementary Fig. 1) was: *n* = 12, range: 45–84, mean (standard deviation): 65.8 (14.2); this distribution is well in keeping with the range for individuals with higher-functioning ASD or near-clinical aggregations of autistic traits. Out of the 21CF/25NCF, there were two CF who only had one point-light run; all other participants had two acceptable runs; all of these participants were included in the full analyses. In sum, CF and NCF groups did not differ in relation to age, cognitive functioning, or quantitative autistic traits (see Statistical Analyses, below; see also, Additional file 1: Supplementary Materials for a discussion of sample characteristics in the present study vs. selected prior literature).

### Statistical analyses

Potential differences between the groups in age, cognitive functioning (IQ; assessed via the Raven's Progressive Matrices), and quantitative autistic traits (SRS score) were tested with the Welch–Satterthwaite corrected *t *test. All statistical analyses of point-light fMRI task data were performed using in-house software programmed in the FIDL language (Research Systems, Inc., Boulder, CO). BOLD activity related to watching scrambled and biological movies was modeled for each participant using the general linear model (GLM) [41]. Rather than assuming a shape for the hemodynamic response function, a value was estimated for 39 time points (21 frames for each 24-s point-light display with an additional 18 frames to estimate a tail-off effect). Baseline and trend terms were also estimated for each GLM. This set of GLMs served as the primary set used for all further analysis. A secondary set of GLMs was also computed, modeling the response to each type of movie with a boxcar regressor for its 24-s duration, and was utilized as a quality check for magnitude effects in each region of interest.

First, we performed hypothesis-driven analyses in 12 regions of interest from Kaiser et al. [26]. For each of the 12 previously described regions, we averaged data within voxels contained within a 15-mm diameter sphere centered at the given coordinates. We used consistent region sizes to maintain consistent signal to noise for all regions adapted from the previous study. Region-wise repeated measures ANOVAs (rm-ANOVAs) were performed to test for meaningful differences in time courses between groups and movies. Factors were included for movie type (scrambled and biological), group (CF and NCF), and time (39 frames). The use of time was to see differences between the timecourses including their shapes. This approach does not make assumptions about the shapes or duration of the responses; we felt that this was important in this case. A Bonferroni multiple comparisons correction of 12 was applied to these tests to control for false-positive rate.

**Fig. 1:**
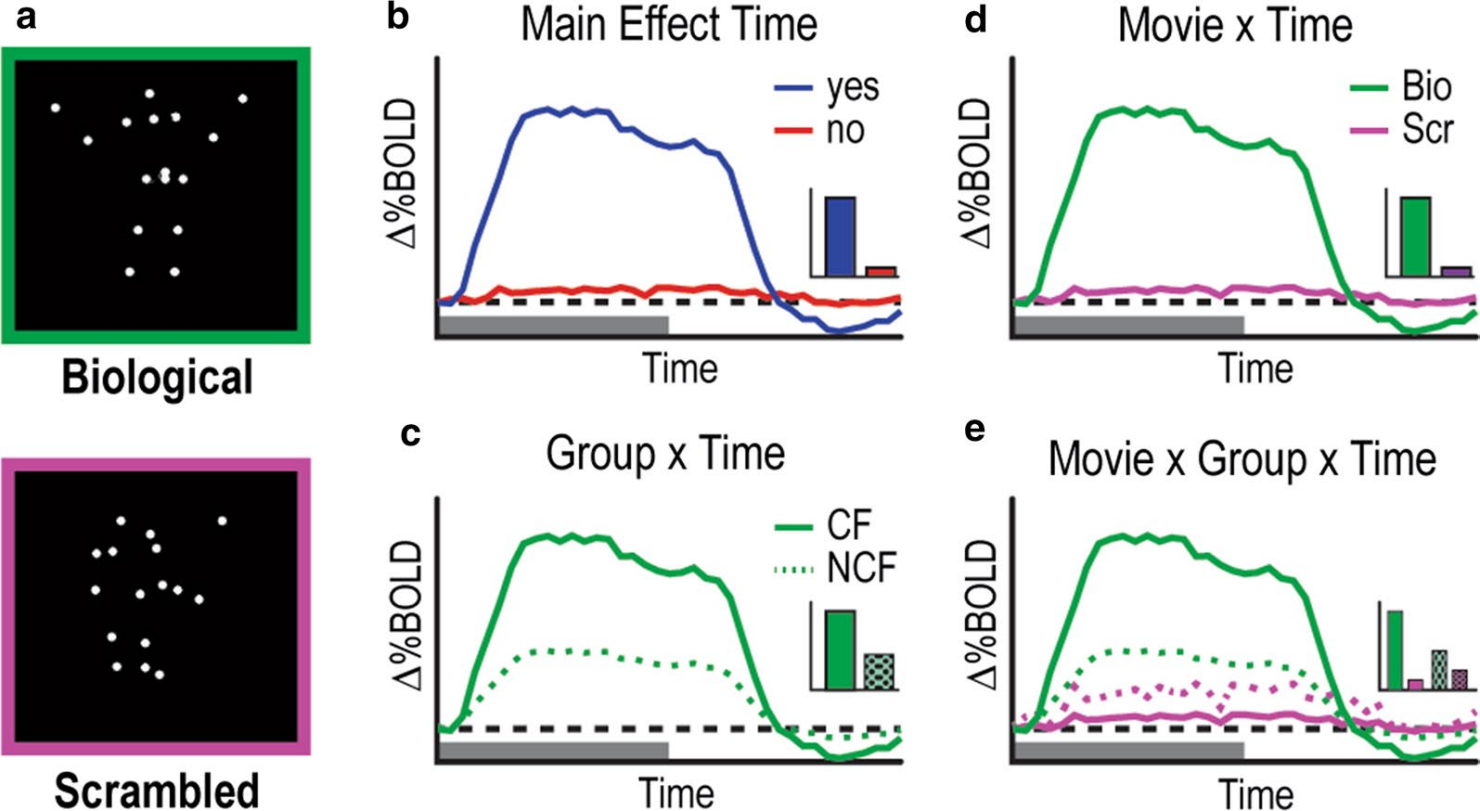
Experimental and analytical design. **a** Single frames of the biological and scrambled point-light movies. **b** The first-order response of interest is a main effect of time associated with the stimulus paradigm. Time courses are highlighted with magnitude differences in inset. Additional higher-order differences across the data include a **c** group by time effect, **d** a movie by time effect, and **e** a movie by group by time effect

In a second data-driven analysis, a whole-brain voxelwise rm-ANOVA on the primary set of GLMs was performed, again including factors for movie type (scrambled and biological), group (CF and NCF), and time (39 frames). The four statistical images of interest that were produced by this full-factor rm-ANOVA included a main effect of time (MET) image, a movie × time (MT) interaction image indicating where time courses for scrambled movies differed significantly from time courses for biological movies (across CF and control participants), a group × time (GT) interaction image indicating where time courses between each group differed significantly across biological and scrambled movies, and a movie × group × time (MGT) image indicating regions with significant variance over time that reflect differential effects of movie across group (Fig. 1). Regions of interest were extracted from the MGT, MT, and GT images using an in-house peak-finding algorithm (https://readthedocs.org/projects/4dfp/). First, the interaction images were smoothed using a 4-mm Gaussian kernel. Then a Monte Carlo correction for multiple comparisons was performed within these voxelwise analyses to model a null distribution of cluster sizes. Peaks greater than 10 mm from another peak and with a minimum *Z*-score of 3.5 were considered, and clusters of at least 24 contiguous voxels in size when masked by the Monte Carlo-corrected image were retained for region-wise analyses. We visually inspected the brain activity in all regions found through these statistical methods and omitted and removed any regions from further analyses whose time-courses were noisy and had maximum magnitude less than 0.1% blood oxygenation level (BOLD) signal change.

Third (to perform analyses restricted to clusters of brain voxels sensitive in some way to movie and/or group effects), region-wise rm-ANOVAs were performed on the resultant multiple sets of significant regions from the above brain-wide rm-ANOVA. Specifically, for each of the MT and GT regions extracted from the whole-brain ANOVA, a post hoc rm-ANOVA was performed to identify any additional significant effects. This post hoc testing in each region was to evaluate for specific regions with multiple significant effects (e.g., an MT or MGT effect in a GT region from the voxelwise rm-ANOVA) in a manner with greater statistical power than in the whole-brain, voxelwise, full-factor analysis.

In the present study, we focused our analyses on a subset of regions including (1) those exhibiting MGT effects and (2) regions exhibiting both movie and group type effects (i.e., GT regions with MT effects and MT regions with GT effects). All described regions passed a Bonferroni multiple comparisons correction for all effect types reaching significance. In our final analyses, for all regions in the study, we tested for a linear relationship between the biological > scrambled contrast and subjects’ SRS scores. Data visualizations were conducted in Matlab. Cortical views include a coloring underlay based on previously described functional parcellation [44].

## Results

### Analyses focused on previously described regions

**Fig. 2:**
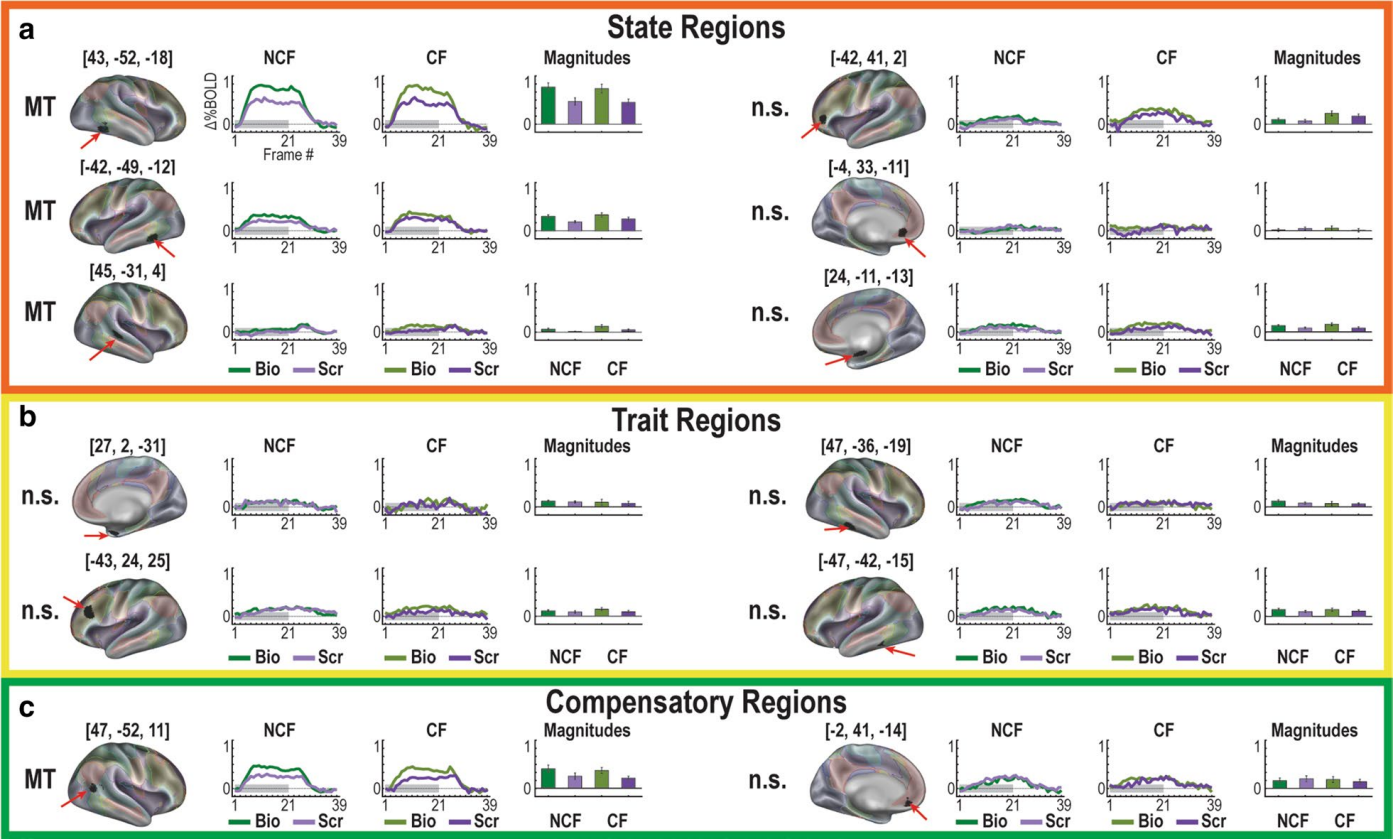
Hypothesis-driven analyses. Previously described regions [26] reported to exhibit differential activation patterns characteristic of (**a**) state, (**b**) trait, and (**c**) compensatory responses were assessed via ANOVA. Significant contrasts of biological > scrambled motion Movie × Time (MT) responses are apparent in four regions. However, no Movie × Group × Time effects were exhibited between the carrier females (CF) and non-carrier females (NCF; n.s., not significant). Gray bar in time courses represents the movie duration

The primary hypothesis-driven analyses of this study were to assess brain responses within previously described regions [26] that exhibited differential responses to passive viewing of point-light movies of biological and scrambled motion (Fig. 2). Four of the previously described regions exhibited significant movie × time effects (MT, Fig. 2a): right posterior superior temporal sulcus (rpSTS, [45, − 31, 4], *n* = 251 voxels, *z* = 5.42, *p* < 10^–4^), right fusiform gyrus ([43, − 52, − 18], *n* = 251 voxels, *z* = 19.25, *p* < 10^–4^), left fusiform gyrus ([− 42, − 49, − 12], n = 251 voxels, *z* = 12.45, *p* < 10^–4^), and right posterior temporal sulcus ([47, − 52, 11], *n* = 251 voxels, *z* = 14.88, *p* < 10^–4^, Fig. 2c). Group differences were not observed in any of these regions. The remaining eight regions did not exhibit any significant effects (see Additional file 1: Supplementary Table 1 for full statistics).

### Brain-wide analyses

**Fig. 3:**
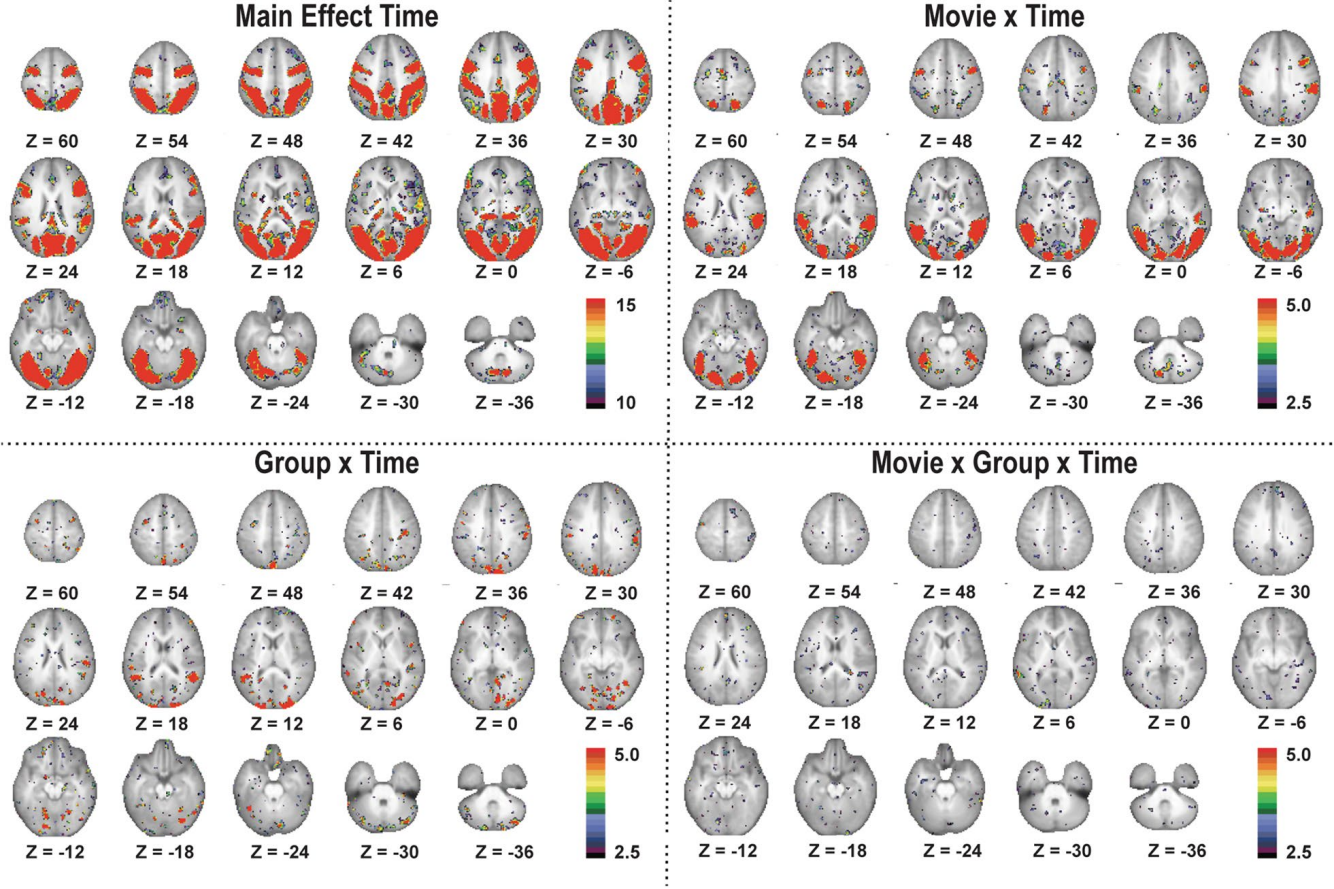
Results of voxelwise ANOVA for each level of analyses. Colorbars reflect voxelwise effect sizes. While much of the brain is modulated by the stimulus paradigm (Main Effect Time map), the differential effect of the movie type (Movie × Time map) is primarily localized to regions associated with visual, social, and attention processes. The interactions of group with the paradigm (Group × Time map) and the full interaction map (Movie × Group × Time) reveal smaller focal regions throughout the brain

**Fig. 4:**
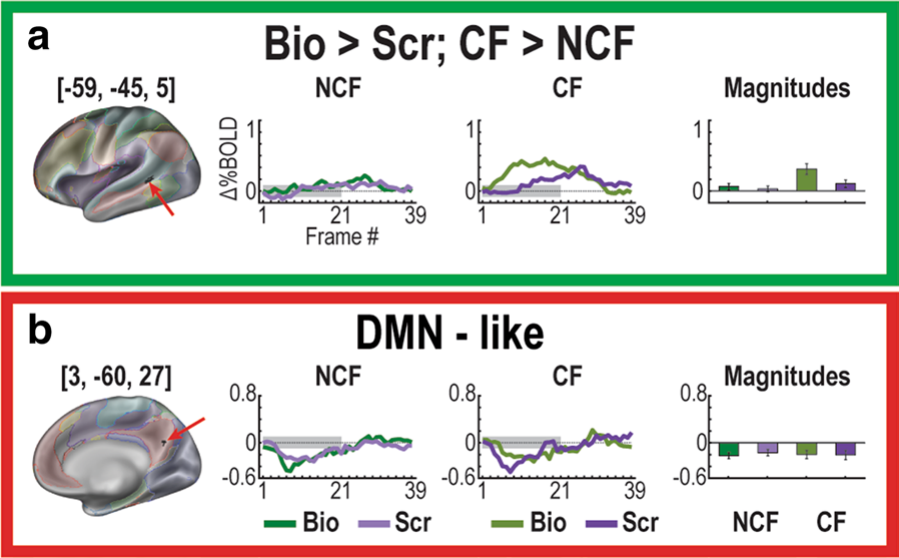
The two regions with Movie × Group × Time effects. **a** A region on left posterior middle temporal sulcus (− 59, − 45, 5) exhibits a stronger response for biological than scrambled motion and has a significantly stronger contrast in the CF than NCF group. **b** One additional region (3, − 60, 27) is also significant but exhibits default-like (negative) characteristics in its response

We then conducted an exploratory brain-wide rm-ANOVA to describe any additional regions containing significant GT, MT, and MGT effects (Fig. 3). Extracting regions from each interaction image resulted in 59 regions from the GT image, 40 regions from the MT image, and two regions from the MGT image. This brain-wide rm-ANOVA analysis uncovered a total of two distinct regions with MGT effects (Fig. 4). One region, in left posterior superior temporal sulcus (lpSTS, [− 59, − 45, 5], *n* = 63 voxels, *z* = 6.53, *p* < 10^–4^) exhibited stronger responses to biological motion than scrambled motion in the CF group, with the NCF group showing no real distinctions between movie types in its responses (Fig. 4a). The second region that exhibited a significant MGT effect was in right posterior cingulate cortex (rPCC, [3, − 60, 27], *n* = 27 voxels, *z* = 5.38, *p* < 10^–4^) and exhibited default-like (i.e., negative) responses to both movie conditions in each group (Fig. 4b).

**Fig. 5:**
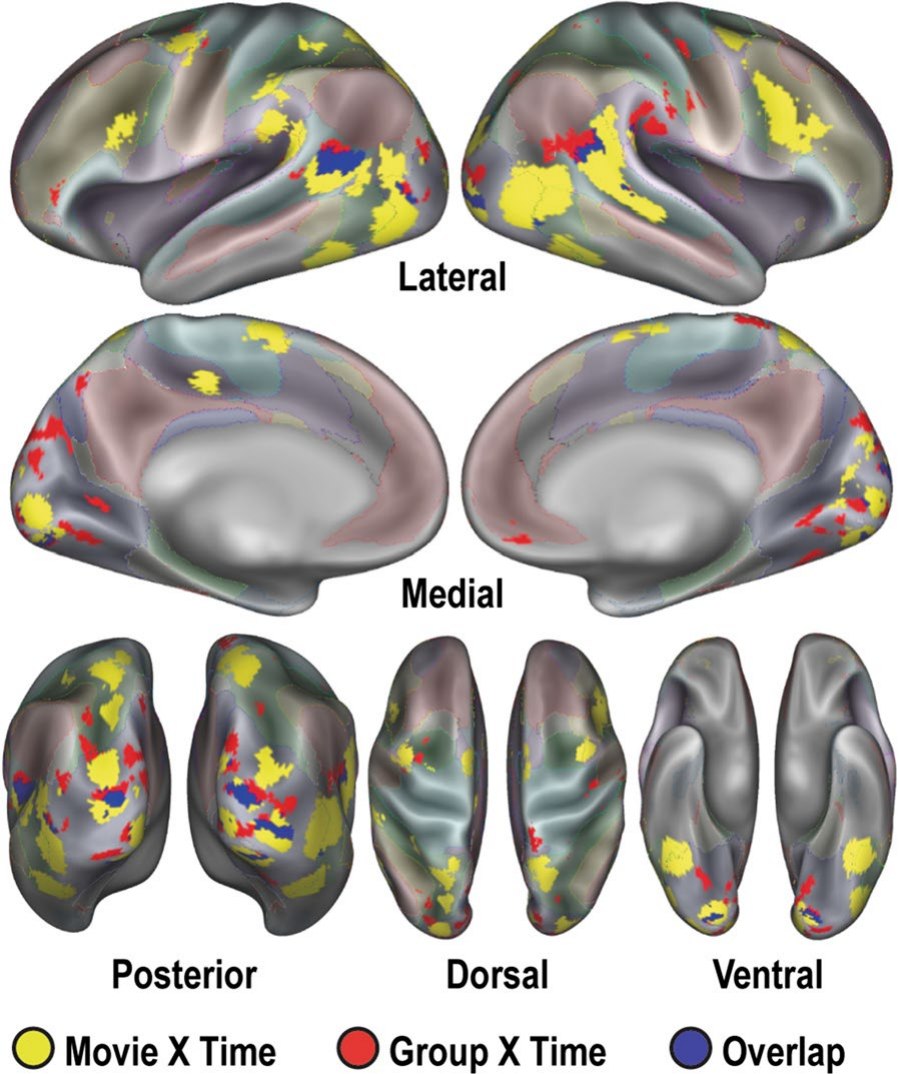
Brain-wide ANOVA reveals robust Movie × Time and Group × Time regions. Regions with significant MT (yellow) effects cover bilateral aspects of occipital lobes, posterior temporal areas, medial parietal, fusiform, and dorso-lateral prefrontal regions. Regions with significant GT (red) effects populate mainly the medial and lateral occipital lobes and medial parietal lobes. Multiple regions of overlap (blue) are apparent in occipital and temporal areas

We also examined regions exhibiting significant effects for either movie × time (MT) or group × time (GT) (Fig. 5). Regions with significant MT effects (yellow) extend over bilateral aspects of occipital lobes, posterior temporal areas, medial parietal, fusiform, and dorso-lateral prefrontal regions. Regions with significant GT (red) effects were found mainly in the medial and lateral occipital lobes and medial parietal lobes. Multiple regions of overlap (blue) are apparent in occipital and temporal areas.

### Additional region-wise post hoc analyses

**Fig. 6:**
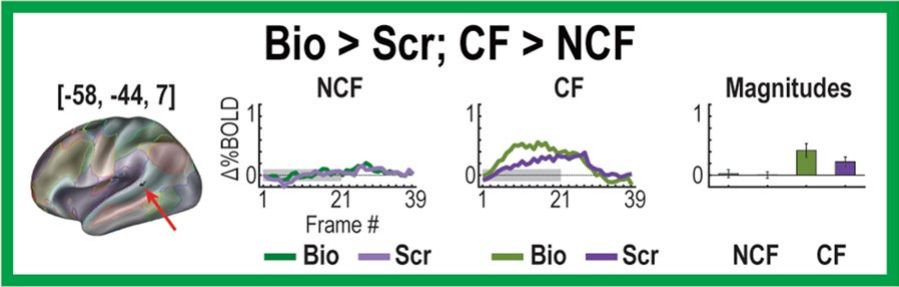
Additional region with Movie × Group × Time effect from post-hoc tests. A region on left posterior middle temporal sulcus (− 58, − 44, 7) exhibits a stronger response for biological than scrambled motion and has a significantly stronger contrast in the CF than NCF group

To characterize more fully the rich set of responses to this paradigm, next we performed post hoc rm-ANOVAs on all GT and MT regions to identify other meaningful effects (i.e., identifying MGT effects in GT-derived regions, etc.). This analysis uncovered one additional MGT region in lpSTG, ([− 58, − 44, 7], *n* = 27 voxels, *z* = 4.21, *p* < 10–4), that exhibited stronger responses to biological motion than scrambled motion in the CF group, with the NCF group showing no real distinctions between movie types in its responses (Fig. 6). This region was from the original set of GT regions. None of the MT regions had significant MGT effects in these analyses after correcting for multiple comparisons.

**Fig. 7:**
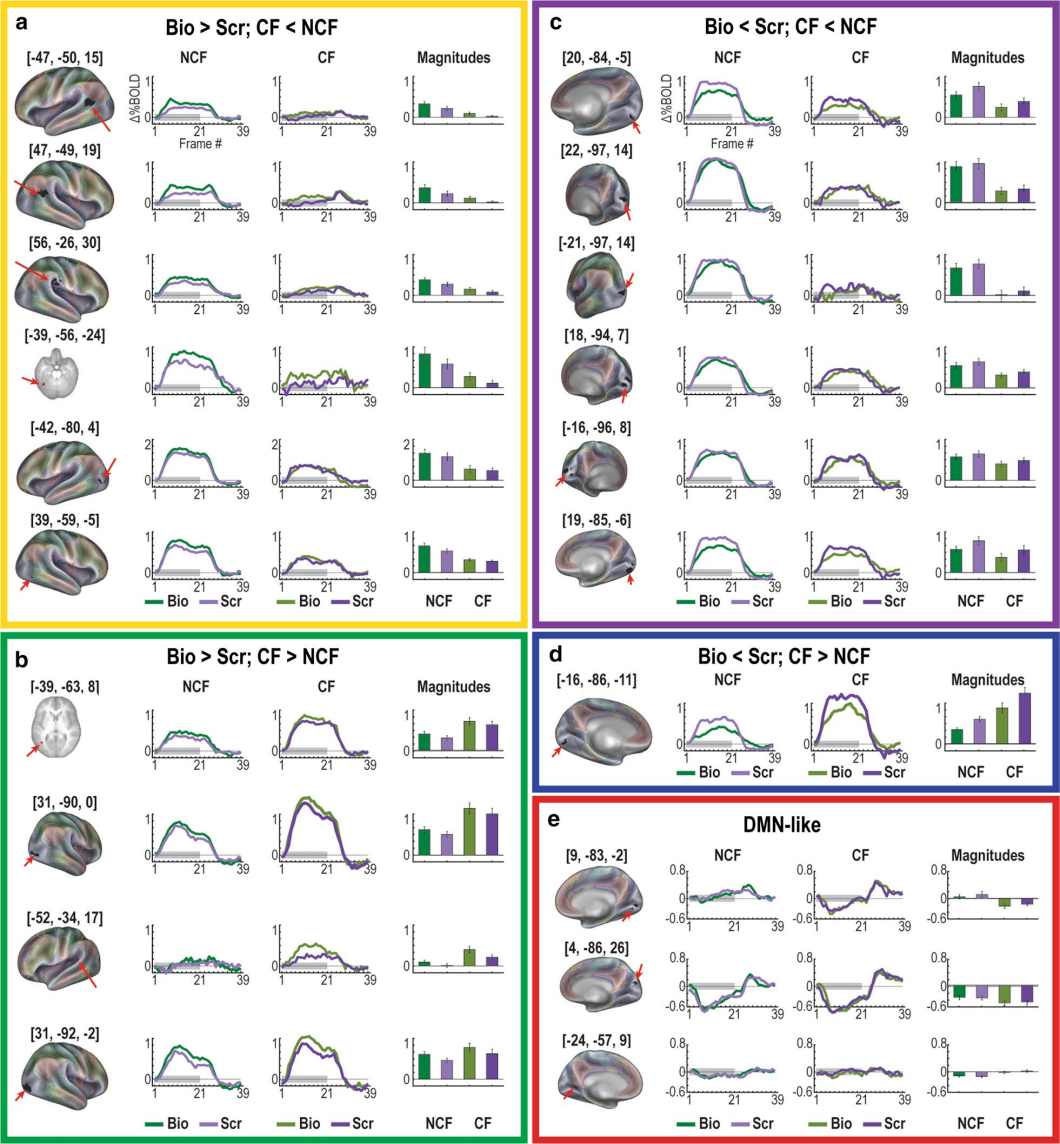
Discovery findings highlight regions with both GT and MT—but not MGT—effects. **a** These regions show significant biological > scrambled effects with stronger responses in NCF. **b** These regions show significant biological > scrambled effects with stronger responses in CF. **c**, **d** Significant MT and GT effects were also exhibited in other regions where biological < scrambled. These regions were entirely located in primary visual areas with stronger effects in the NCF group for most (**c**), and stronger effects in the CF group in one region (**d**). **e** Three additional regions in occipital lobe exhibited significant MT and GT effects with default-like responses

Post hoc testing in each of the MT and GT regions revealed 20 regions (Fig. 7) with both GT and MT (but not the full MGT interaction) with five types of responses (see Additional file 1: Supplementary Table 2 for full statistics). First, six regions exhibited responses with significant biological > scrambled responses with stronger activations in NCF (Fig. 7a). Second, four regions exhibited significant biological > scrambled with stronger responses in CF (Fig. 7b). Third, seven regions exhibited significant MT and GT effects but where biological < scrambled (Fig. 7c, d). These regions were entirely located in primary visual areas with stronger activation in the NCF group for most (Fig. 7c), and stronger effects in the CF group in one region (Fig. 7d). Last, three additional regions in occipital lobe exhibited significant MT and GT effects with default-like responses (Fig. 7e). It should be strongly noted that the combination of MT and GT effects does not imply MGT interactions (meaning that, instead, both biological and non-biological motion followed similar patterns across groups). This is true both statistically and to visual inspection of timecourse effects themselves.

Correlation analyses between the contrast of biological vs scrambled motion in all regions assessed in this study revealed no significant associations with cognitive ability or social responsiveness in either the CF or NCF group, nor as a whole.

## Discussion

Herein, we aimed to investigate whether previously reported neural signatures of familial risk of ASD—elicited by passive viewing of point light displays of biological motion—were related to carrier status for elevation in family genetic risk of ASD. Our study design was optimized to isolate genetic risk factors: we strongly enriched our sample for adult carrier females (CF), with control, non-carrier females (NCF) matched for sex, age, parity, and cognitive/behavioral variation. Although we identified numerous contrasts between CF and NCF cohorts, we failed to replicate an array of specific brain responses that have previously been reported to differentiate siblings of ASD probands from unrelated controls (cf. [26]). Our results make two fundamental contributions to the literature. First, markers of brain function contrasts may index carrier status for possible silent transmission of genetic risk of ASD in the absence of measurable behavioral phenotypic indicators. Second, these results highlight the subtleties of a growing body of the literature utilizing biological motion perception paradigms to study neural and behavioral endophenotypes of ASD. Indeed, results with these paradigms may depend intimately on age and sex structure of the cohorts and on the specific type of biological motion paradigm used [45, 46].

Regarding neural signatures of elevated genetic susceptibility, our analyses revealed numerous group × time effects (GT; n = 59 distinct regions), indicating pervasive differences in neural responding to point light stimuli between CF and NCF cohorts. These effects were observed across regions implicated in biological motion (e.g., pSTS [47]), non-biological (coherent) motion (e.g., regions along the dorsal stream, including the temporo-parietal-occipital junction, V3/V3A, and V6 [48]), and signal integration (e.g., TPJ, which has also been implicated in biological motion perception [49–52]). We observed few movie × group × time effects (MGT; n = 3), revealing that, in general, group differences were not modulated by the biological content of stimuli. Group effects may instead reflect subtle variations in visual attention and motion processing related to genetic risks for ASD—risks which, generally, do not manifest as subclinical ASD behaviors. These results are consistent with a recent meta-analysis [45] in which the magnitude of motion-processing deficits in ASD was invariant to movie type (i.e., coherent motion vs. biological motion), suggestive of domain-general—rather than specifically social—motion processing deficits. Domain-general deficits could arise due to atypical visual processing along the dorsal stream [53–55], such as we observed in the temporo-parietal-occipital junction, V3/V3A, and V6; they could also arise due to atypical integration of sensory signals later in processing [56–58], such as we observed in the TPJ. Signal integration features prominently in Bayesian and predictive coding theories of ASD, and atypicalities thereof have been posited to account for core ASD symptoms, as well as characteristic ASD behaviors [59–61]. Relative to well-characterized biological > scrambled motion contrasts [62–65], less work has sought to characterize brain regions that preferentially respond to scrambled > biological motion. One recent study to do so reported significant results for scrambled > biological motion in occipital and prefrontal cortex [66]; we also observed significant results for scrambled > biological motion in occipital cortical regions (Fig. 6). It is worth noting that group differences of neural signatures of elevated genetic susceptibility have also been observed within the amygdala and fusiform gyrus in an analogous study of face processing in parents of children with ASD [67]. 

Regarding biological motion as a candidate endophenotype of ASD, we acknowledge that our study lacked an ASD clinical group. As such, we cannot rule out the possibility that biological motion effects are specific to ASD rather than indicative of genetic risk of ASD. Current evidence for biological motion effects in ASD is mixed. Although there are many reports of reduced sensitivity to biological motion in individuals with ASD [68–70], there are likewise a number of reported null findings [71–73]. Recent meta-analyses aimed at clarifying these discrepancies concluded that biological motion effects in ASD are weak, non-specific, and highly conditional on experimental design [45, 46, 74]. Experimental design may vary with respect to task features (e.g., spatially scrambled vs. phase-scrambled motion [68, 70]), sample characteristics (e.g., infants vs. adults [75, 76]), and response set (e.g., preferential looking vs. reaction time [76, 77]). To minimize variability, the present study used the identical experimental stimuli as Kaiser et al. [26]. Additionally, to reduce analytical assumptions, we opted for straightforward statistical tests of main effects and interactions. Notwithstanding, our results largely failed to replicate specific brain responses previously reported to differentiate siblings of ASD probands from unrelated controls [26], perhaps due to sampling differences motivated by research goals (see Additional file 1: Supplementary Materials). The lack of clear replication with respect to biological motion effects highlights the need for additional research examining experimental conditions under which biological motion may be used as an endophenotypic marker in ASD.

## Limitations

Our study specifically tested for group differences in brain function during passive viewing of PLDs to understand better the mechanism(s) by which genetic liability for ASD can be carried in clinically unaffected individuals, including silently transmitting parents. A limitation of our study was that we were unable to successfully eye-track all participants, which prevented us from being able to control for potential differences in eye gaze position. Indeed, studies frequently report robust differences in eye gaze between children with ASD and typically developing controls [78, 79], cautioning against a straightforward interpretation of group effects from task-based designs. A number of recent studies shed further light on gaze patterns in ASD, suggesting that these patterns are under remarkable genetic control [36], emerge in infancy [35], and persist across development [78]. In the absence of eye-tracking data, we note that CF and NCF cohorts both exhibited comparable biological/non-biological motion contrasts.

## Conclusions

These observations offer deeper insight into the brain activation effects of increased genetic susceptibility to ASD among clinically unaffected members of ASD-affected families. The possibility of a parsimonious effect of sex—and therefore a convergent neural signature of its effect in modulating phenotypic expression of inherited liability—arises from the observation that, along the autism spectrum and across its many genetic causes, the symptom structure of the condition is unitary in nature. Thus, disparate symptoms might arise from shared neural mechanisms [80, 81] that are uniquely vulnerable to disruption early in life—less among females, more among males—in individuals who inherit ASD susceptibility [5, 82, 83]. In the present study, we failed to replicate an array of specific brain responses that were reported to differentiate siblings of ASD probands from unrelated controls in prior research. We did, however, observe robust differential responses to point-light stimuli in CF vs. NCF cohorts, raising the possibility that neural responding to global (rather than specifically biological) motion may constitute a neural signature of enhanced genetic susceptibility to ASD. This highly unique, female sample highly enriched for family genetic loading of ASD risk enabled discovery of multiple potential targets for future investigation of the effects of inherited ASD susceptibility on brain development and function.

## Supplementary information

**Additional file 1.** Supplementary information containing a figure detailing the predigrees of the CF group, tables containing details on regions of interest for the hypothesis-driven analyses and brain-wide exploratory analyses, and a brief discussion on differences in cohorts in this and the Kaiser 2010 study.

## Data Availability

The datasets used and/or analyzed during the current study are available from the corresponding author on reasonable request.

## Abbreviations

ABCL: Adult behavior checklist
ASD: Autism spectrum disorder
BOLD: Blood oxygenation level-dependent signal
CF: Carrier female
fMRI: Functional magnetic resonance imaging
FPE: Female protective effect
GLM: General linear model
GT: Group by time effect
MET: Main effect of time
MGT: Movie by group by time effect
MT: Movie by time effect
NCF: Non-carrier female
PLD: Point-light displays
RMS: Root mean square
SRS: Social responsivity scale
TD: Typically developing
US: Unaffected sibling
WUSM: Washington University School of Medicine
