# Supplementary material for: Brain function distinguishes female carriers and non-carriers of familial risk for autism
Source: Mol Autism. 2020 Oct 20;11:82. doi: 10.1186/s13229-020-00381-y (PMC7574590; doi:10.1186/s13229-020-00381-y)
Supplement: Supplementary file 1 — Additional file 1. Supplementary information containing a figure detailing the predigrees of the CF group, tables containing details on regions of interest for the hypothesis-driven analyses and brain-wide exploratory analyses, and a brief discussion on differences in cohorts in this and the Kaiser 2010 study. [file 13229_2020_381_MOESM1_ESM.docx]

**Supplementary Figure 1**

| 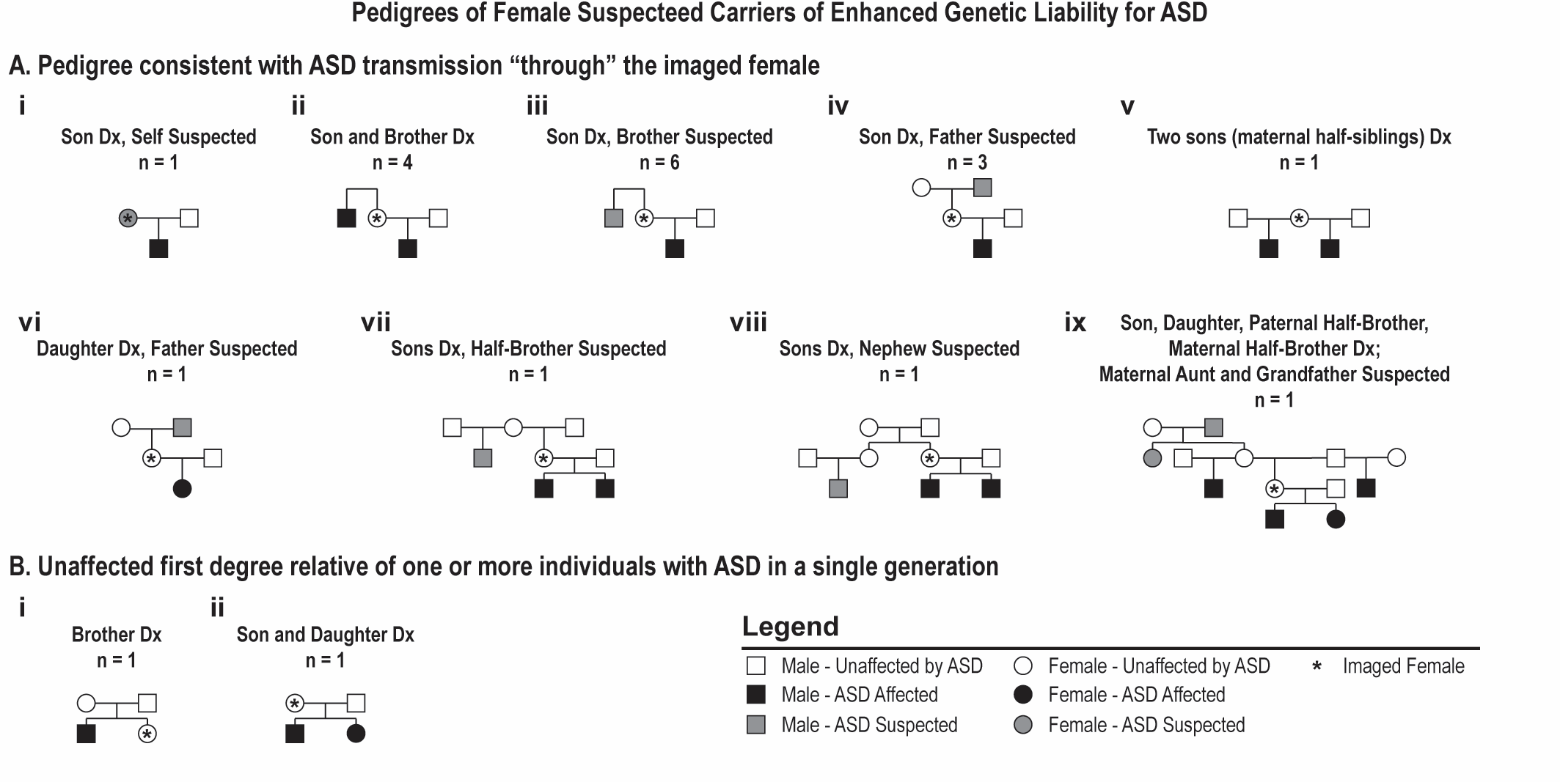 |
| --- |
| **Supplementary Figure 1 \| Pedigrees of the adult carrier female (CF) group.** Pedigrees in the study contained 11 distinct patterns that fit into two categories. **A** Strong enrichment for carrier status on the basis of a pedigree consistent with intergenerational transmission of ASD through the imaged subject; this was the category for most participants in the CF group. **B** Modest enrichment for carrier status; this characterized two unaffected female subjects with one or more first degree relatives with ASD in a single generation. |

**Supplementary Table 1**

| **Region** | **Proposed neural signature** | **Coordinates (Talairach, *x y z*)** | **Effects observed** | **Size (2 mm^3^ voxels)** |
| --- | --- | --- | --- | --- |
| Left ventrolateral prefrontal cortex | State | –42 41 2 | *none* | 251 |
| Ventromedial prefrontal cortex | State | –4 33 –11 | *none* | 251 |
| Right posterior temporal sulcus | State | 45 –31 4 | MT | 251 |
| Right amygdala | State | 24 –11 –13 | *none* | 251 |
| Right fusiform gyrus | State | 43 –52 –18 | MT | 251 |
| Left fusiform gyrus | State | –42 –49 –12 | MT | 251 |
| Right inferior temporal sulcus | Trait | 27 2 –31 | *none* | 251 |
| Left dorsolateral prefrontal gyrus | Trait | –43 24 25 | *none* | 251 |
| Right fusiform gyrus | Trait | 47 –36 –19 | *none* | 251 |
| Left fusiform gyrus | Trait | –47 –42 –15 | *none* | 251 |
| Right posterior temporal sulcus | Compensatory | 47 –52 11 | MT | 251 |
| Ventromedial prefrontal cortex | Compensatory | –2 41 –14 | *none* | 251 |

MT, movie by time effect.

**Supplementary Table 2**

| **Region source image** | **Coordinates (Talairach, *x y z*)** | **Effects observed** | **Size (2 mm^3^ voxels)** |
| --- | --- | --- | --- |
| MGT | –59 –45 5 | MGT | 63 |
| MGT | 3 –60 27 | MGT | 27 |
| GT | –58 –44 7 | GT, MGT, MT | 27 |
| GT | –16 –86 –11 | GT, MT | 90 |
| GT | 20 –84 –5 | GT, MT | 92 |
| GT | 22 –97 14 | GT, MT | 213 |
| GT | –47 –50 15 | GT, MT | 213 |
| GT | 47 –49 19 | GT, MT | 232 |
| GT | –39 –63 8 | GT, MT | 50 |
| GT | 31 –90 0 | GT, MT | 80 |
| GT | 56 –26 30 | GT, MT | 200 |
| GT | –39 –56 –24 | GT, MT | 35 |
| GT | –42 –80 4 | GT, MT | 84 |
| GT | –21 –97 14 | GT, MT | 178 |
| GT | 39 –59 –5 | GT, MT | 74 |
| GT | –52 –34 17 | GT, MT | 38 |
| GT | 9 –83 –2 | GT, MT | 124 |
| GT | –24 –57 9 | GT, MT | 50 |
| MT | 18 –94 7 | MT, GT | 332 |
| MT | –16 –96 8 | MT, GT | 372 |
| MT | 31 –92 –2 | MT, GT | 213 |
| MT | 19 –85 –6 | MT, GT | 434 |
| MT | 4 –86 26 | MT, GT | 118 |

MGT, movie by group by time effect. MT, move by time effect. GT, group by time effect.

**Supplementary Materials**

Cohort age and sex profiles differed between the present study and Kaiser et al. (2010). Whereas Kaiser et al. focused analyses on n=20 unaffected siblings (n=11 female) and n=17 typically developing participants (n=5 female)^1^, the present study focused exclusively on females (n = 21 carrier females, n = 25 non-carrier females) so as not be confounded by potential sex effects. Previous analyses of sex effects were conducted in typically developing participants and therefore have not evaluated sex-by-genetic-liability interaction effects^2^. In addition to sex profiles, the age range in the present study (24-64 years) was older relative to Kaiser et al. (4.6 – 16.9 years), rendering variation due to developmental effects less likely. Both this study and Kaiser et al. (2010) matched groups based on SRS scores, though it is possible our study did not observe the correlation with SRS due to the smaller range exhibited by our cohorts.

**References**

1. Kaiser MD, Hudac CM, Shultz S, Lee SM, Cheung C, Berken AM, et al. Neural signatures of autism. Proceedings of the National Academy of Sciences of the United States of America. 2010;107(49):21223-8.
2. Martino D, Madhusudan N, Zis P, Cavanna AE. An introduction to the clinical phenomenology of Tourette syndrome. Int Rev Neurobiol. 2013;112:1-33.
